# 81. Reducing Unnecessary Blood Cultures Through Diagnostic Stewardship

**DOI:** 10.1093/ofid/ofab466.283

**Published:** 2021-12-04

**Authors:** Sujeet Govindan, Luke Strnad

**Affiliations:** Oregon Health & Science University, Portland, Oregon

## Abstract

**Background:**

At our institution, we learned the frequency of blood cultures was sometimes being changed from “Once” to “Daily” without a defined number of days. We hypothesized this led to unnecessary blood cultures being performed.

**Methods:**

Over a 3 month period from 12/6/2019-3/6/2020, we retrospectively evaluated the charts of patients who had a blood culture frequency changed to “Daily”. We evaluated if there was an initial positive blood culture within 48 hours of the “Daily” order being placed and the number of positive, negative, or “contaminant” sets of cultures drawn with the order. Contaminant blood cultures were defined as a contaminant species, present only once in the repeat cultures, and not present in initial positive cultures.

**Results:**

95 unique orders were placed with 406 sets of cultures drawn from 89 adults. ~20% of the time (17 orders) the order was placed without an initial positive blood culture. This led to 62 sets of cultures being drawn, only 1 of which came back positive. 78/95 orders had an initial positive blood culture. The most common initial organisms were *Staphylococcus aureus* (SA) (38), *Candida sp* (10), Enterobacterales sp (10), and coagulase negative staphylococci (7). 43/78 (55%) orders with an initial positive set had positive repeat cultures. SA (26) and *Candida sp* (8) were most common to have positive repeats. Central line associated bloodstream infections (CLABSI) were found in 5 of the orders and contaminant species were found in 4 of the orders. 54% of the patients who had a “Daily” order placed did not have positive repeat cultures. The majority of the cultures were drawn from Surgical (40 orders) and Medical (35 orders) services. Assuming that SA and *Candida* sp require 48 hours of negative blood cultures to document clearance and other species require 24 hours, it was estimated that 51% of the cultures drawn using the "Daily" frequency were unnecessary. Cost savings over a year of removing the "Daily" frequency would be ~&14,000.

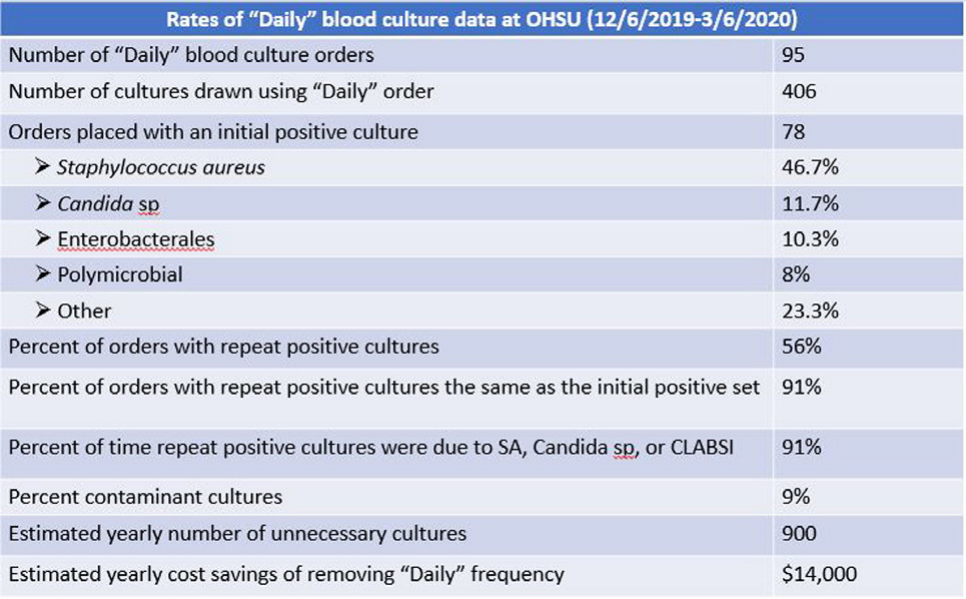

Data from "Daily" blood culture orders drawn at Oregon Health & Science University from 12/6/2019-3/6/2020

**Conclusion:**

Unnecessary blood cultures are drawn when the frequency of blood cultures is changed to "Daily". Repeat blood cultures had the greatest utility in bloodstream infections due to *SA or Candida* sp, and with CLABSI where the line is still in place. These results led to a stewardship intervention to change blood culture ordering at our institution.

**Disclosures:**

**All Authors**: No reported disclosures

